# Association between the triglyceride-glucose index and the risk of post-stroke depression

**DOI:** 10.3389/fpsyt.2026.1877775

**Published:** 2026-08-25

**Authors:** Xiaohang Su, Jueyu Zhao, Yifan Li, Xin Li

**Affiliations:** Department of Neurology, The Affiliated Hospital of Qingdao University, Qingdao, China

**Keywords:** acute ischemic stroke, insulin resistance, post-stroke depression, triglyceride-glucose index, metabolic biomarkers

## Abstract

**Background:**

Post-stroke depression (PSD) is a common complication after stroke that affects prognosis. The triglyceride-glucose (TyG) index, a surrogate marker of insulin resistance, is associated with cerebrovascular diseases, but its relationship with PSD remains unclear. This study investigated the association between TyG index and PSD risk in first-ever acute ischemic stroke (AIS) patients.

**Methods:**

We enrolled 398 consecutive first-ever AIS patients and calculated baseline TyG index. PSD was assessed using the 17-item Hamilton Depression Rating Scale at 3 months post-stroke. Logistic regression, restricted cubic splines (RCS), and threshold effect models were used to evaluate the association and dose-response relationship.

**Results:**

At 3 months, 126 patients (31.66%) developed PSD. The PSD group had significantly higher TyG index than the non-PSD group (9.03[8.34–9.34] vs. 8.56[8.17–9.05], P < 0.001). After adjusting for sex, NIHSS score, LDL-C, and HDL-C, TyG index remained independently associated with PSD (OR = 1.64, 95% CI: 1.28–2.09, P < 0.001). Patients in the highest TyG quartile had a significantly increased PSD risk compared with the lowest quartile (OR = 3.40, 95% CI:1.73–6.68, P < 0.001). RCS analysis revealed a non-linear relationship, with a turning point at 8.02 identified by threshold effect analysis; above this value, PSD risk increased significantly (OR = 1.97, 95% CI:1.43–2.72, P < 0.001). Subgroup analyses showed significant interactions in patients with diabetes (P for interaction = 0.046) and hyperlipidemia (P for interaction = 0.026).

**Conclusions:**

Elevated TyG index is independently associated with increased PSD risk in a non-linear manner. A TyG index ≥ 8.02 may serve as a risk threshold for PSD, and this association appears more pronounced in patients with diabetes or hyperlipidemia.

## 1 Introduction

Stroke is a leading cause of death and long-term disability worldwide (1), with acute ischemic stroke (AIS) accounting for approximately 70%–80% of all stroke cases (2). Advances in acute management and secondary prevention strategies have significantly improved survival rates among stroke patients. However, post-stroke complications have increasingly emerged as critical determinants of long-term prognosis. Post-stroke depression (PSD) is one of the most common neuropsychiatric sequelae in AIS patients, characterized by persistent low mood, anhedonia, loss of interest, and in some cases, sleep disturbances, appetite changes, diminished concentration, and suicidal ideation (3, 4). Epidemiological studies indicate that approximately 18%–33% of stroke survivors develop depressive symptoms within 3–6 months after stroke onset (3). PSD not only adversely affects neurological recovery and quality of life but is also strongly associated with increased risks of stroke recurrence and mortality. Therefore, early identification of individuals at high risk for PSD is of paramount clinical importance for improving post-stroke outcomes.

The pathogenesis of PSD remains incompletely understood but is widely believed to involve a complex interplay among neurotransmitter imbalance, inflammatory responses, hypothalamic-pituitary-adrenal (HPA) axis dysfunction, and metabolic disturbances (5, 6). In recent years, the role of metabolic dysregulation in post-stroke neuropsychiatric sequelae has garnered increasing attention. Insulin resistance (IR), a core component of metabolic syndrome, is not only involved in the development of various metabolic diseases but also closely associated with cardiovascular and cerebrovascular disorders (7, 8). IR is defined as reduced sensitivity of target tissues to physiological insulin levels, thereby impairing glucose metabolism regulation (9). Previous studies have suggested a bidirectional relationship between IR and depression (10, 11), but its role in PSD remains to be further elucidated. Some studies have found that higher levels of IR assessed by the homeostasis model assessment of insulin resistance (HOMA-IR) upon admission are associated with an increased risk of PSD in stroke populations (12). However, HOMA-IR relies on fasting insulin measurement, which is not a routine clinical test, limiting its widespread application in clinical practice.

The triglyceride-glucose (TyG) index, a simple indicator calculated from fasting triglyceride and glucose levels, has been validated as a reliable surrogate marker of IR (13). In recent years, the TyG index has been extensively utilized in research on metabolic syndrome, cardiovascular disease, and cerebrovascular disease. Accumulating evidence suggests that a higher TyG index may be associated with the occurrence of depressive symptoms (14, 15). However, most existing studies have focused on the general population or patients with diabetes, with limited research specifically targeting patients with first-ever AIS. Whether an independent association exists in this specific population and the nature of the potential dose-response relationship remain unclear.

Therefore, this study aimed to investigate the association between the TyG index and the risk of PSD in patients with first-ever AIS, and to further explore potential nonlinear associations and threshold effects, with the goal of providing a novel reference indicator for early risk assessment of PSD.

## 2 Manuscript formatting

### 2.1 Materials and methods

This was a single-center retrospective cohort study that consecutively enrolled 398 patients with first-ever AIS who were hospitalized in the Department of Neurology at the Affiliated Hospital of Qingdao University between June 2024 and March 2025.

**Figure 1:**
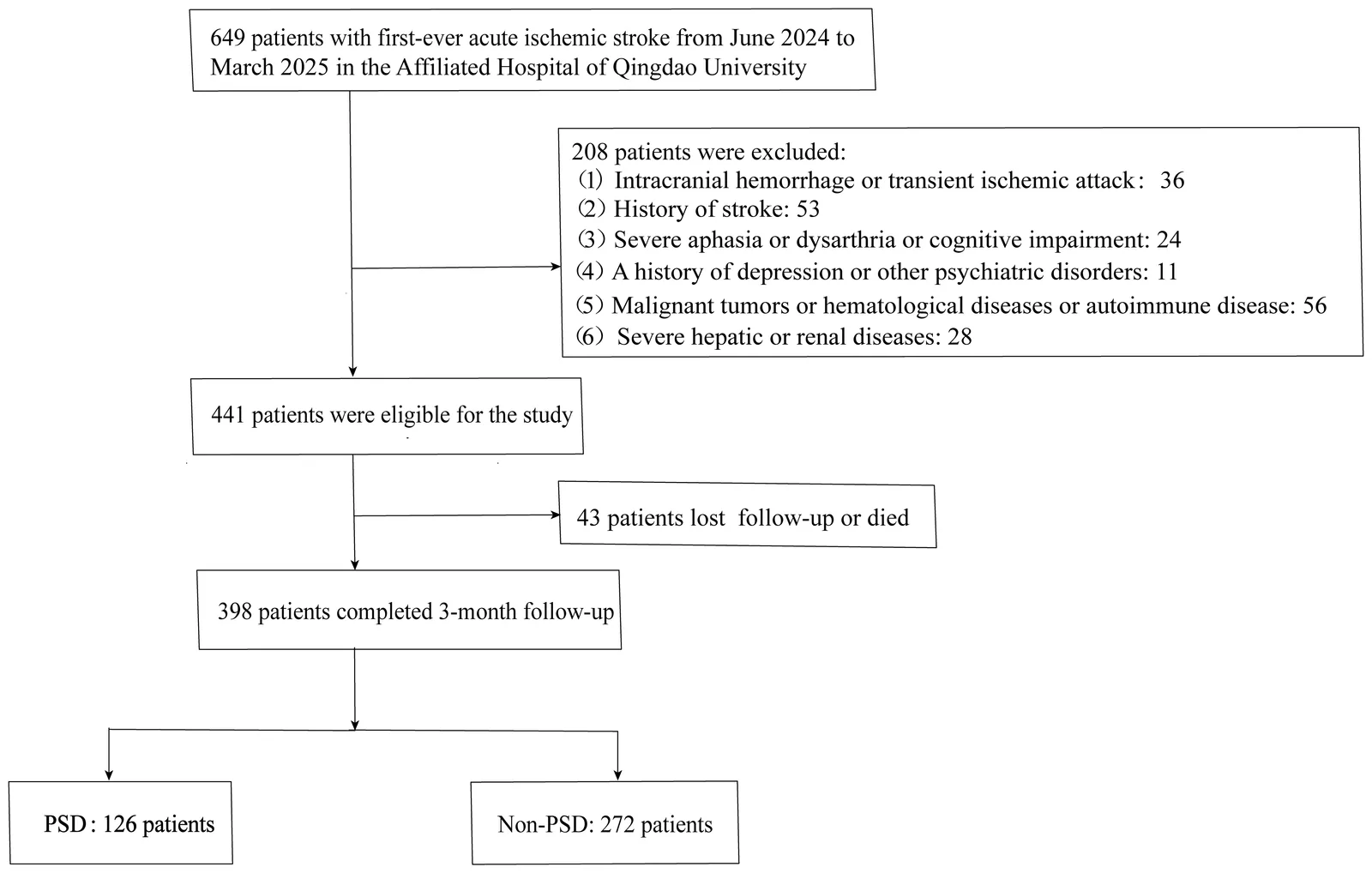
Flow chart of subject selection.

The inclusion criteria were as follows: (1) age between 18 and 80 years; (2) admission within 7 days after stroke onset; and (3) diagnosis of AIS confirmed by computed tomography (CT) or magnetic resonance imaging (MRI) after admission. The exclusion criteria were as follows: (1) intracranial hemorrhage or transient ischemic attack (TIA); (2) previous history of stroke; (3) severe aphasia, dysarthria, disturbance of consciousness, or cognitive impairment that prevented completion of the depression assessment; (4) a previous history of depression or other psychiatric disorders; (5) concomitant malignant tumors, hematologic diseases, or autoimmune diseases; (6) severe hepatic or renal dysfunction (**Figure 1**).

### 2.2 Clinical data collection

Demographic data, including age, sex, marital status, and education level, were collected for all patients. Vascular risk factors were also recorded, including smoking history, alcohol consumption, hypertension, diabetes mellitus, hyperlipidemia, coronary heart disease, and atrial fibrillation. Stroke severity was assessed within 24 hours of admission by trained neurologists using the National Institutes of Health Stroke Scale (NIHSS).

### 2.3 Laboratory measurements and calculation of TyG index

Venous blood samples were collected from all patients within 24 hours of admission after an overnight fast of at least 8 hours. All laboratory tests were performed using standardized procedures and uniform operating protocols. Laboratory measurements included fasting blood glucose (FBG), alanine aminotransferase (ALT), aspartate aminotransferase (AST), homocysteine (HCY), total cholesterol (TC), triglycerides (TG), high-density lipoprotein cholesterol (HDL-C), low-density lipoprotein cholesterol (LDL-C), and serum creatinine (Scr). The TyG index was calculated using the following formula: TyG index = ln [TG (mg/dL) × FBG (mg/dL)**/**2] (16).

### 2.4 Assessment of PSD

All patients were followed up at 3 months after stroke onset. Depressive symptoms were assessed using the 17-item Hamilton Depression Rating Scale (HAMD-17). A HAMD-17 score **>**7 was defined as PSD, while patients with HAMD-17 scores **≤**7 were classified into the non-PSD group.

### 2.5 Statistical analysis

All statistical analyses were performed using SPSS version 27.0 and R software version 4.4.3. The Kolmogorov–Smirnov test was used to assess the normality of continuous variables. Normally distributed variables were expressed as mean ± standard deviation (SD) and compared using the independent-samples t-test. Non-normally distributed data were presented as median (interquartile range), and comparisons were made using the Mann-Whitney U test. Categorical variables were expressed as frequencies and percentages and compared using the Pearson χ² test or Fisher**’**s exact test. Binary logistic regression models were used to evaluate the association between the TyG index and PSD. Univariate logistic regression was first performed, and variables with a *P*-value < 0.05 were entered into the multivariate logistic regression model. Due to the high collinearity between TyG and TG levels, TG was not included in the multivariate model. The TyG index was then categorized into quartiles (Q1–Q4) based on the distribution of the entire study population (cutoffs at the 25th, 50th, and 75th percentiles). The lowest quartile (Q1) served as the reference group. Odds ratios (ORs) and 95% confidence intervals (CIs) were calculated for each quartile, and a trend test (*P* for trend) was performed by treating quartiles as a continuous variable in the regression model to evaluate the linear dose-response relationship between the TyG index and PSD. To explore the potential nonlinear relationship between the TyG index and PSD, restricted cubic spline (RCS) analysis with four knots was performed. A piecewise linear regression model was subsequently used for threshold effect analysis to identify possible inflection points. In subgroup analyses, stratification variables—including sex, smoking history, alcohol consumption history, hypertension, diabetes, hyperlipidemia, coronary heart disease, and atrial fibrillation—were prespecified based on clinical relevance and previous literature. These variables represent common stroke comorbidities and demographic/lifestyle factors that may modify the association between insulin resistance and depression. Subgroup analyses were conducted to evaluate the consistency of the association between the TyG index and PSD across different populations. The likelihood ratio test was used to assess interaction effects between each subgroup variable and the TyG index to determine whether effect modification existed. All statistical tests were two-tailed, and P < 0.05 was considered statistically significant.

## 3 Results

### 3.1 Comparison of baseline characteristics between PSD and non-PSD groups

During the study period, a total of 649 patients with AIS were screened, of whom 441 met the inclusion criteria. During the 3-month follow-up period, 43 patients (9.75%) were excluded due to loss to follow-up (including refusal to participate, invalid contact information, death, or other reasons), and a total of 398 patients completed the follow-up and were included in the final statistical analysis. At 3 months after stroke, 126 patients (31.66%) developed PSD.

The baseline characteristics of patients in the PSD and non-PSD groups are presented in **Table 1**. Compared with the non-PSD group, the PSD group had a higher proportion of females (P = 0.010) and more severe neurological deficits (P < 0.001). TG, LDL-C, and TyG index were significantly higher in the PSD group (all P < 0.001), whereas HDL-C levels were significantly lower (P < 0.001).

**Table 1:** Baseline Characteristics of PSD and Non-PSD Groups.

Variables	Total(n=398)	PSD(n=126)	Non-PSD (n=272)	P-value
Age(years)	66.00(59.00-73.00)	65.50(59.45-73.00)	67.00(59.25-72.00)	0.499
Sex, n(%)				0.010
Male	260(65.33%)	71(56.35%)	189(69.49%)	
Female	138(34.67%)	55(43.65%)	83(30.51%)	
Education, n(%)				0.297
Primary school and below	165(41.46%)	57(45.24%)	108(39.71%)	
Junior high school and above	233(58.54%)	69(54.76%)	164(60.29%)	
Marital status, n(%)				0.712
Married	347(87.19%)	111(88.10%)	236(86.76%)	
Others	51(12.81%)	15(11.90%)	36(13.24%)	
Smoking history, n(%)	175(43.97%)	48(38.10%)	127(46.69%)	0.108
Drinking history, n(%)	183(45.98%)	49(38.89%)	134(49.26%)	0.053
Hypertension, n(%)	294(73.87%)	95(75.40%)	199(73.16%)	0.637
Diabetes, n(%)	103(25.88%)	40(31.75%)	63(23.16%)	0.069
Hyperlipidemia, n(%)	144(36.18%)	51(40.48%)	93(34.19%)	0.225
Coronary heart disease, n(%)	42(10.55%)	18(14.29%)	24(8.82%)	0.099
Atrial fibrillation, n(%)	22(5.53%)	11(8.73%)	11(4.04%)	0.057
NIHSS score	2.00(0.00-5.00)	3.00(1.00-6.00)	2.00(0.00-4.00)	<0.001
Cr(μmol/L)	59.20(50.23-68.80)	57.60(47.80-68.05)	59.45(50.80-69.00)	0.245
ALT(U/L)	20.00(16.00-25.52)	21.00(16.00-26.67)	20.00(16.23-25.00)	0.564
AST(U/L)	24.00(20.00-27.00)	23.00(20.00-27.00)	24.00(20.00-28.00)	0.425
HCY(μmol/L)	8.80(5.95-11.00)	8.85(4.79-10.97)	8.70(6.47-11.10)	0.419
TG(mmol/L)	1.54(0.97-3.95)	2.52(0.95-5.16)	1.38(0.98-2.60)	<0.001
TC(mmol/L)	3.85(1.61-4.78)	3.84(1.32-4.82)	3.87(2.91-4.73)	0.501
LDL-C(mmol/L)	2.64(2.12-2.95)	2.69(2.12-3.21)	2.62(2.13-2.79)	<0.001
HDL-C(mmol/L)	1.19(1.00-1.45)	1.10(0.98-1.20)	1.28(1.02-1.56)	<0.001
TyG index	8.64(8.23-9.16)	9.03(8.34-9.34)	8.56(8.17-9.05)	<0.001

NIHSS, National Institutes of Health Stroke Scale; Cr, creatinine; ALT, alanine aminotransferase; AST, aspartate aminotransferase; HCY, homocysteine; TG, triglyceride; TC, total cholesterol; LDL-C, low-density lipoprotein cholesterol; HDL-C, high-density lipoprotein cholesterol; TyG index, triglyceride-glucose index.

### 3.2 Logistic regression analysis of factors associated with PSD

Variables with P < 0.05 in the univariate analysis were included in the multivariate logistic regression model, and the results are shown in **Table 2**. Because TG is a component of the TyG index, it was excluded from the multivariate model to avoid multicollinearity. After adjusting for sex, NIHSS score, LDL-C, and HDL-C, the TyG index remained an independent risk factor for PSD (adjusted OR = 1.64, 95% CI: 1.28–2.09, P < 0.001).

**Table 2:** Univariate and Multivariate Logistic Regression Analysis for PSD.

Variables	Crude OR(95% CI)	P-value	Adjusted OR(95% CI)	P-value
Female	1.76(1.14-2.73)	0.011	1.56(0.96-2.54)	0.076
NIHSS score	1.16(1.08-1.24)	<0.001	1.14(1.06-1.23)	<0.001
TG	1.38(1.23-1.55)	<0.001	–	–
HDL-C	0.22(0.11-0.43)	<0.001	0.28(0.14-0.58)	<0.001
LDL-C	2.19(1.61-2.99)	<0.001	2.35(1.67-3.30)	<0.001
TyG index	2.06(1.42-2.98)	0.002	1.64(1.28-2.09)	<0.001

Sex, LDL-C, HDL-C, and NIHSS score were adjusted.

### 3.3 Association between TyG index and PSD

**Figure 2:**
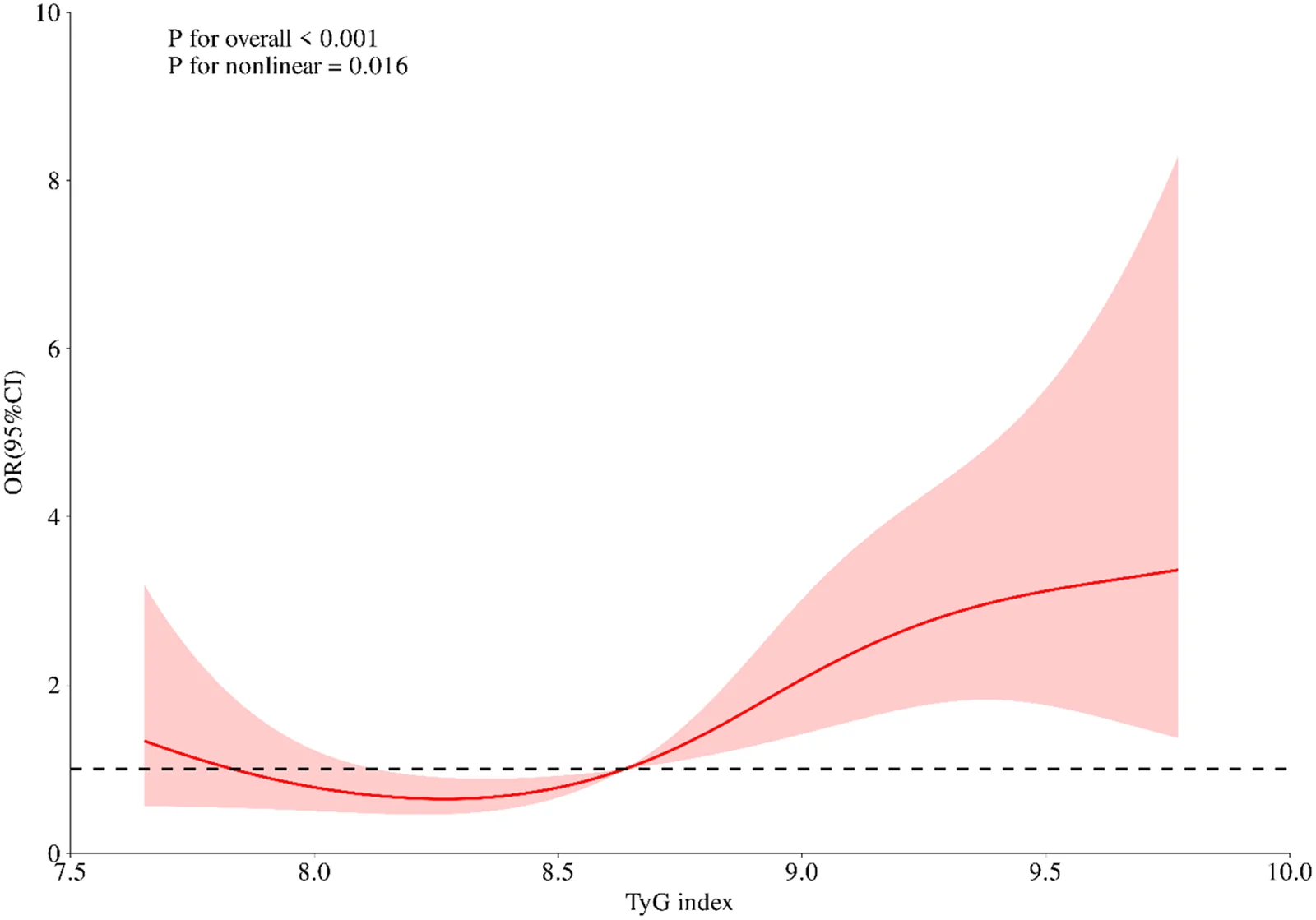
The association between the TyG index and PSD.

The association between the TyG index and PSD is shown in **Table 3**. When analyzed as a continuous variable, the crude model (Model 1) showed that each 1-unit increase in the TyG index was associated with a 1.54-fold higher risk of PSD (OR = 1.54, 95% CI: 1.23–1.92, P < 0.001). After adjusting for potential confounders, including sex, age, hypertension, diabetes, NIHSS score, LDL-C, and HDL-C (Model 3), the TyG index remained significantly associated with PSD (adjusted OR = 1.61, 95% CI: 1.26–2.06, P < 0.001), indicating that an elevated TyG index was an independent risk factor for PSD. When the TyG index was categorized into quartiles, compared with the lowest quartile group (Q1: < 8.23), patients in the highest quartile group (Q4: ≥9.17) had a significantly increased risk of PSD (crude OR = 2.88, 95% CI: 1.58–5.25, P < 0.001; adjusted OR = 3.40, 95% CI: 1.73–6.68, P < 0.001). RCS analysis revealed a nonlinear dose–response relationship between the TyG index and PSD risk (**Figure 2**). Segmented linear regression threshold analysis identified a turning point of 8.02 for the TyG index. When TyG was < 8.02, the risk of PSD was not significantly increased (OR = 0.19, 95% CI: 0.02–1.74, P = 0.142). However, when TyG ≥ 8.02, the risk of PSD increased significantly (OR = 1.97, 95% CI: 1.43–2.72, P < 0.001) (**Table 4**).

**Table 3:** Association between TyG index and PSD.

Variables	Model 1		Model 2		Model 3	
	OR (95%CI)	P-value	OR (95%CI)	P-value	OR (95%CI)	P-value
TyG index(continuous)	1.54(1.23-1.92)	<0.001	1.48(1.18-1.85)	<0.001	1.61(1.26-2.06)	<0.001
TyG index quartiles						
Q1 (<8.23)	Reference		Reference		Reference	
Q2 (8.23–8.64)	0.92(0.48-1.76)	0.802	0.91(0.47-1.77)	0.791	0.99(0.48-2.06)	0.986
Q3 (8.64–9.17)	1.23(0.66-2.29)	0.524	1.13(0.60-2.13)	0.715	1.49(0.74-3.01)	0.264
Q4 (≥ 9.17)	2.88(1.58-5.25)	<0.001	2.67(1.45-4.92)	0.002	3.40(1.73-6.68)	<0.001
P for trend	<0.001		0.002		<0.001	

Model 1: Crude model.

Model 2: Age, sex, hypertension, and diabetes were adjusted.

Model 3: Further adjusted for LDL-C, HDL-C, and NIHSS score.

**Table 4:** Threshold Effect Analysis of TyG Index on PSD Risk.

PSD	OR(95%CI)	P
TyG index		
Model 1:Fitting by the standard linear model	1.61(1.26-2.06)	<0.001
Model 2:Fitting by the two-piecewise linear model		
Inflection point	8.02	
<8.02	0.19 (0.02-1.74)	0.142
≥8.02	1.97 (1.43-2.72)	<0.001
Log-likelihood ratio test		0.034

Age, sex, hypertension, diabetes, LDL-C, HDL-C, and NIHSS score were adjusted.

### 3.4 Subgroup analysis and interaction test

**Figure 3:**
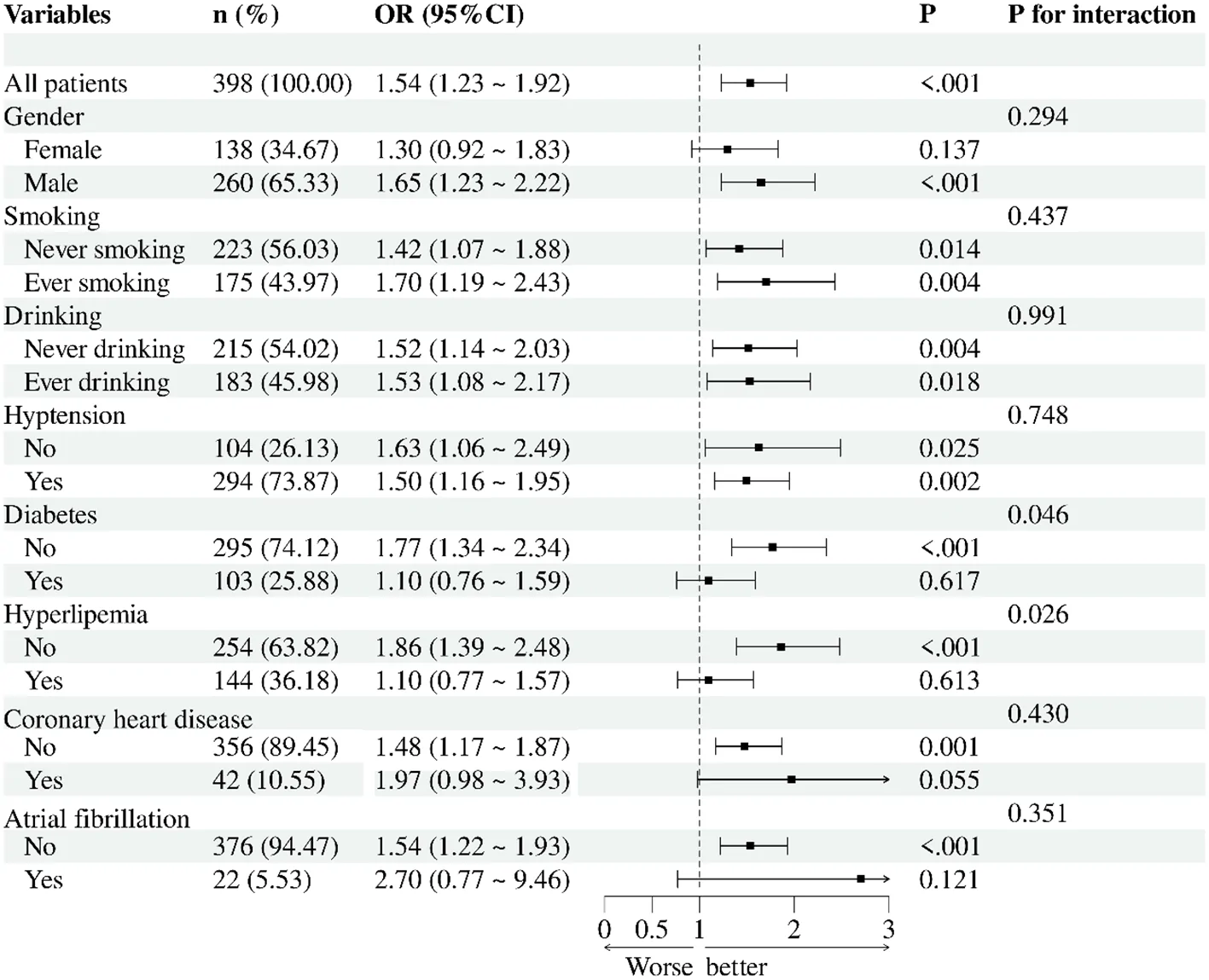
Subgroup analysis.

Subgroup analysis were conducted according to sex, smoking status, drinking status, hypertension, diabetes, hyperlipidemia, coronary heart disease, and atrial fibrillation to evaluate the potential influence of these factors on the association between the TyG index and PSD. The positive association between the TyG index and PSD remained consistent across most subgroups (all P for interaction > 0.05). However, significant interactions were observed in the diabetes (P for interaction = 0.046) and hyperlipidemia (P for interaction = 0.026) subgroups (**Figure 3**).

## 4 Discussion

The present retrospective cohort study involving 398 patients with first-ever AIS investigated the association between the TyG index and the risk of PSD. The results demonstrated that an elevated TyG index was significantly associated with an increased risk of PSD, and this association remained independent after adjusting for potential confounders, including sex, stroke severity, and lipid parameters. Furthermore, RCS analysis revealed a nonlinear dose–response relationship between the TyG index and PSD risk, with a potential threshold identified at 8.02. Subgroup analysis also indicated significant interactions between the TyG index and diabetes as well as hyperlipidemia. These findings suggest that the TyG index, as a simple and low-cost surrogate marker of IR, has promising clinical utility for early identification and risk stratification of individuals at high risk of PSD.

PSD is the most common and significant neuropsychiatric sequela of stroke (17), characterized by anhedonia, low mood, loss of interest, and feelings of worthlessness (18). Previous studies have reported that the incidence of PSD ranges from 11% to 41% (19), which is broadly consistent with the 31.66% observed in the present study. PSD not only severely affects patients’ emotional well-being and quality of life but also delays neurological recovery, reduces rehabilitation adherence, and increases the risk of recurrent stroke and mortality. Accumulating evidence suggests that PSD is not merely a psychological reaction, but rather the result of a complex interplay of neurobiological alterations, inflammatory responses, neurotransmitter imbalances, and metabolic disturbances (20, 21). Therefore, identifying objective biomarkers that can predict the risk of PSD is of great importance for early intervention and improving stroke prognosis.

IR is a core pathophysiological feature of various chronic metabolic diseases (22) and a significant risk factor for cerebrovascular disease (23). Previous studies have demonstrated that IR contributes to the development and progression of atherosclerosis not only by inducing endothelial dysfunction, lipid metabolism disorders, and chronic inflammation (24), but is also closely associated with neuropsychiatric disorders such as cognitive decline, dementia, and depression (25, 26). A meta-analysis involving 240,704 participants revealed that patients with depression exhibited higher levels of insulin resistance and peripheral insulin concentrations compared with healthy controls (10). Currently, although the hyperinsulinemic-euglycemic clamp technique is considered the gold standard for assessing insulin sensitivity, its clinical application is limited by its complexity, invasiveness, and time-consuming nature. The HOMA-IR, calculated based on fasting blood glucose and insulin levels, is relatively simpler but remains susceptible to various physiological and metabolic factors, with its accuracy and stability still subject to certain limitations (27). Therefore, identifying simple, cost-effective, and reliable surrogate markers for IR is of considerable clinical significance.

As a simple surrogate marker calculated based on FBG and TG levels, the TyG index has been widely used to assess IR due to its convenient accessibility, low cost, and good reproducibility (28). An elevated TyG index reflects a state of systemic metabolic disturbance. In recent years, numerous studies have confirmed that the TyG index is closely associated with atherosclerosis, cardiovascular disease, and metabolic syndrome (29, 30), and is also significantly correlated with the risk of depression (31, 32). Moreover, a nonlinear association between the TyG index and depression has been observed in the hypertensive population in the United States (33). However, studies specifically investigating the relationship between TyG and PSD remain relatively limited. Therefore, this study aimed to explore the association between TyG levels and the risk of depression in patients with first-ever AIS, in order to fill the existing research gap and provide a novel theoretical basis for the early identification and intervention of depression in this high-risk population.

Our results showed that the TyG level was significantly higher in the PSD group than in the non-PSD group, and the TyG index was independently and positively associated with PSD. After dividing TyG into quartiles, patients with a TyG ≥ 9.17 had a significantly increased risk of PSD (adjusted OR = 3.40, 95% CI: 1.73–6.68, P < 0.001), suggesting that a higher metabolic burden may be closely related to the occurrence of PSD. Notably, RCS analysis revealed a nonlinear relationship between the TyG index and PSD risk. When TyG was ≥ 8.02, the risk of PSD increased significantly (OR = 1.97, 95% CI: 1.43–2.72, P < 0.001), whereas no significant change in risk was observed below this threshold. This threshold effect suggests that there may be a “danger window” for TyG, such that when insulin resistance reaches a certain level, neurobiological changes may rapidly intensify, thereby triggering the onset of depressive symptoms. Compared with previous studies, our work further expands the evidence base for the association between TyG and depression. Cross-sectional studies have reported that elevated TyG levels are significantly associated with increased depression symptom scores in the general population and in patients with diabetes (31, 34), and some studies have also found a positive association between the TyG index and the risk of depression in the elderly population (35), suggesting that IR-related metabolic abnormalities may be involved in the development of depression. However, most of these studies were cross-sectional in design, making it difficult to infer causality. The present study adopted a cohort design and assessed the occurrence of PSD at three months after stroke, which is more logical in terms of temporal sequence and provides stronger evidence for the role of TyG as a predictor of PSD. Moreover, by focusing on patients with first-ever AIS, our study excluded potential confounding from prior stroke or a clear history of psychiatric disorders, thereby enhancing the specificity of the results.

The role of IR in the pathogenesis of PSD involves multiple complex biological pathways. IR impairs cerebral glucose uptake and utilization, thereby compromising the synthesis and release of mood-related neurotransmitters, including serotonin (5-HT), dopamine (DA), and norepinephrine (NE). IR also promotes the release of pro-inflammatory cytokines such as IL-6 and TNF-α (36), which not only directly disrupt the function of emotion-regulating brain regions (37, 38), but also activate indoleamine-2,3-dioxygenase (IDO). This shifts tryptophan metabolism from 5-HT synthesis toward the kynurenine pathway, reducing cerebral 5-HT production and generating neurotoxic metabolites such as quinolinic acid, which overactivates NMDA receptors and induces excitotoxic injury (39, 40). Furthermore, IR and inflammation form a vicious cycle: chronic inflammation exacerbates IR (41), while IR-activated tissue-resident macrophages secrete TNF-α and IL-1β, which further impair insulin signaling and amplify both local and systemic IR (42). Stroke itself acts as a potent inflammatory event, releasing damage-associated molecular patterns that activate microglia and infiltrating immune cells (43). This, combined with chronic low-grade inflammation associated with IR, creates a “double-hit” insult that heightens immune responsiveness to post-stroke injury signals, while elevated pro-inflammatory cytokines further aggravate IR, synergistically promoting PSD.

In the state of IR, antioxidant defense mechanisms are impaired, and when combined with the excessive production of reactive oxygen species during post-stroke ischemia-reperfusion, oxidative damage is markedly amplified. Studies have shown that serum malondialdehyde (MDA) levels are positively correlated with HAMD scores in stroke patients, and MDA ≥ 2.898 nmol/ml independently predicts PSD (44). Using NHANES data, Li et al. reported that the oxidative balance score (OBS), a composite measure of overall antioxidant status, was inversely associated with PSD risk (OR = 0.31) (45). Sánchez-García et al. further confirmed that in mice fed a high-fat diet, MDA levels in white adipose tissue increased significantly before the onset of peripheral IR, whereas high-carbohydrate diet-induced cerebral IR was accompanied by decreased glutathione peroxidase (GSH-Px) activity and elevated protein carbonyl (PCO) levels in the cerebral cortex (46).

IR also induces hyperactivation of the hypothalamic-pituitary-adrenal axis, leading to sustained elevation of cortisol, which exerts neurotoxic effects on hippocampal neurons and exacerbates depressive symptoms (47). As a powerful stressor, stroke may further amplify the interaction between IR and HPA axis dysregulation, thereby increasing the risk of PSD. Stroke activates the HPA axis through both direct stress signaling and indirect pro-inflammatory pathways, resulting in excessive glucocorticoid production. Elevated glucocorticoid levels contribute to PSD pathogenesis by impairing hippocampal neurons, downregulating brain-derived neurotrophic factor expression, promoting microglial activation, and disrupting neurotransmitter metabolism (48). Clinically, markers of HPA axis activity, such as plasma cortisol levels and peak cortisol response, have been shown to correlate positively with the HOMA-IR index in elderly patients with depression (49). Collectively, these findings suggest that HPA axis dysregulation may serve as a key bridging mechanism linking IR to the development of PSD, likely through convergent stress-related and metabolic pathways.

Subgroup analyses in this study showed that the positive association between TyG and PSD remained consistent across subgroups of sex, smoking history, drinking history, hypertension, coronary heart disease, and atrial fibrillation, with no significant interactions observed (all P for interaction > 0.05). This suggests that TyG, as a predictive marker, has good stability and generalizability across most populations and is not limited to specific subgroups. This finding has important implications for clinical practice, because the AIS patient population is heterogeneous, and the utility of a marker would be limited if it were only effective in specific subgroups. Notably, significant interactions were observed in the subgroups of diabetes (P for interaction = 0.046) and hyperlipidemia (P for interaction = 0.026). In non-diabetic individuals, the association between TyG and PSD was stronger (OR = 1.77, 95% CI: 1.34–2.34), whereas in diabetic individuals, this association was not statistically significant (OR = 1.10, 95% CI: 0.76–1.59). Similarly, in non-hyperlipidemic individuals, TyG was significantly and positively associated with PSD (OR = 1.86, 95% CI: 1.39–2.48), whereas no significant association was observed in the hyperlipidemic group (OR = 1.10, 95% CI: 0.77–1.57). Several mechanisms may underlie this phenomenon. First, diabetes and hyperlipidemia themselves are IR-related conditions (50, 51), and patients with these comorbidities generally have elevated baseline IR levels. As a surrogate marker of IR, the predictive performance of the TyG index depends on the variability of IR within the population. When the majority of individuals in a subgroup already exhibit IR, the discriminative ability of TyG within that group becomes limited, making it difficult to effectively identify those at higher risk. Second, these patients often receive long-term treatment with glucose-lowering agents (e.g., metformin) and lipid-lowering drugs (e.g., statins), which may exert beneficial effects through improving IR, mitigating inflammation, or providing direct neuroprotection (52, 53). Such pharmacological interventions may partially offset the depressive risk associated with elevated TyG levels, thereby attenuating the observed association between TyG and PSD in these populations. Third, patients with diabetes or hyperlipidemia frequently have more frequent medical contact and psychological support as part of their chronic disease management, which may also contribute to a reduced risk of PSD. These findings suggest that the metabolic comorbidity status of patients should be carefully considered when applying the TyG index for PSD risk stratification. In AIS patients with coexisting diabetes or hyperlipidemia, the predictive value of TyG may be limited, and a comprehensive assessment incorporating other clinical indicators is recommended.

From a clinical perspective, our study has important implications. First, the TyG index, derived from routine laboratory tests, is simple, inexpensive, and reproducible, making it a practical tool with significant potential for clinical translation. Incorporating the TyG index into the initial evaluation of AIS patients may facilitate early identification of individuals at high risk of PSD, enabling timely risk stratification even during the acute stage of stroke. Moreover, metabolic interventions such as improved glycemic control, reduction of triglyceride levels, and lifestyle modifications—including healthy diet and increased physical activity—may not only reduce the risk of stroke recurrence but also potentially lower the incidence of PSD. Future prospective interventional studies are needed to determine whether improving TyG levels can effectively reduce the occurrence of PSD and thereby provide new therapeutic targets for the comprehensive management of AIS patients.

Several limitations of this study should also be acknowledged. First, this was a single-center retrospective cohort study, with all patients enrolled from the Department of Neurology at the Affiliated Hospital of Qingdao University between June 2024 and March 2025. The relatively limited sample source and short enrollment period may introduce selection bias, and potential seasonal variations in baseline characteristics could have influenced the results. Therefore, multicenter prospective studies are warranted to confirm our findings. Second, of the 649 patients initially screened, 43(9.75%) were excluded due to loss to follow-up. Although this attrition rate is within an acceptable range compared to similar studies, we did not perform a comparative analysis between the included and excluded populations. Thus, we cannot completely rule out the possibility of selection bias, and this should be taken into consideration when interpreting the results. Third, the TyG index was measured only at baseline, with no longitudinal assessments during follow-up. Consequently, we were unable to evaluate the relationship between dynamic changes in the TyG index and the long-term risk of PSD. Fourth, the diagnosis of PSD was based on the HAMD-17 scale. Although this instrument is widely used and has well-established reliability and validity, it is still subject to potential interviewer-related bias. Fifth, this study primarily focused on insulin resistance as the core mechanism underlying the association between the TyG index and PSD, and did not explore the potential role of other metabolic comorbidities, such as metabolic dysfunction-associated steatotic liver disease (MASLD), in this relationship. As a common manifestation of systemic metabolic dysfunction, MASLD is often accompanied by chronic low-grade inflammation, endothelial dysfunction, and autonomic imbalance, which may independently or synergistically influence the risk of PSD (54). Future studies are warranted to further evaluate whether coexisting MASLD modifies the association between TyG levels and PSD risk. In addition, despite adjusting for multiple potential confounders, we cannot exclude the influence of unmeasured variables, such as social support, individual coping styles, inflammatory biomarkers, and neuroimaging features. Finally, the study population consisted of patients with first-ever AIS, and whether these findings can be generalized to patients with hemorrhagic stroke or recurrent stroke remains to be further investigated.

## 5 Conclusion

In summary, this study demonstrated that an elevated TyG index is independently associated with an increased risk of post-stroke depression in patients with first-ever acute ischemic stroke. A nonlinear dose–response relationship was observed, with a potential threshold effect at a TyG value of 8.02. Given its simplicity and availability in routine clinical practice, the TyG index may serve as a useful metabolic marker for early identification of patients at high risk of PSD. Further multicenter prospective studies are needed to confirm these findings and explore the underlying mechanisms.

## Data Availability

The original contributions presented in the study are included in the article/Supplementary Material, further inquiries can be directed to the corresponding author/s.
